# ESTIMATED ARTERIAL STIFFNESS, COGNITIVE DECLINE, AND DEMENTIA AMONG MIDDLE-AGED AND OLDER ADULTS

**DOI:** 10.1093/geroni/igad104.3093

**Published:** 2023-12-21

**Authors:** Kevin Heffernan, Janet Wilmoth, Andrew London

**Affiliations:** Syracuse University, Syracuse, New York, United States; Syracuse University, Syracuse, New York, United States; Syracuse University, Maxwell School of Citizenship and Public Affairs, Syracuse, New York, United States

## Abstract

Prior research suggests arterial stiffness may be a useful vascular biomarker that predicts age-related cognitive decline. In this paper, we use the Health and Retirement Study (HRS) to examine the relationship between estimated pulse wave velocity (ePWV), which is an under-analyzed but readily available estimate of arterial stiffness, and cognitive decline. The HRS assesses cognitive function with the Telephone Interview for Cognitive Status (TICS). In this 27-point TICS measure, scores between 7 and 11 indicate cognitively impaired but no dementia (CIND) and scores between 0 and 6 indicate dementia. We modeled the relationship between ePVW and cognitive status change from 2006/2008 through 2018. The findings indicate ePWV increases the odds of having CIND and dementia relative to having no CIND or dementia. In addition, ePWV increases the odds of transitioning into CIND and dementia from a higher cognitive functioning state. The results are robust to controlling for: age, age squared, age cubed, systolic and diastolic blood pressure along with sociodemographic characteristics (gender, race, ethnicity, wealth, income, education), health status (history of cardiovascular disease (CVD,) diabetes, and stroke and related medication use), health behaviors (smoking, physical activity, body mass index), and CVD-related biomarkers (C-reactive protein, cystatin-C, hemoglobin A1c, total cholesterol, high-density lipoprotein (HDL) cholesterol). These findings suggest that ePWV is a simple screening tool and novel biomarker that can be used to monitor vascular cognitive aging and CIND progression in middle-aged and older adults.

